# Development and characterization of polymer-coated liposomes for vaginal delivery of sildenafil citrate

**DOI:** 10.1080/10717544.2016.1247925

**Published:** 2017-02-06

**Authors:** Hanan Refai, Doaa Hassan, Rehab Abdelmonem

**Affiliations:** 1 Department of Pharmaceutics and Industrial Pharmacy, Faculty of Pharmacy, Cairo University, Cairo, Egypt and; 2 Department of Pharmaceutics and Industrial Pharmacy, Faculty of Pharmacy, Misr University for Science and Technology, 6th of October City, Egypt

**Keywords:** Chitosan, histopathological study, *ex vivo* vaginal permeation, polymer-coated liposomes, sildenafil citrate, vaginal administration

## Abstract

Vaginal administration of sildenafil citrate has shown recently to develop efficiently the uterine lining with subsequent successful embryo implantation following *in vitro* fertilization. The aim of the present study was to develop sildenafil-loaded liposomes coated with bioadhesive polymers for enhanced vaginal retention and improved drug permeation. Three liposomal formulae were prepared by thin-film method using different phospholipid:cholesterol ratios. The optimal liposomal formulation was coated with bioadhesive polymers (chitosan and HPMC). A marked increase in liposomal size and zeta potential was observed for all coated liposomal formulations. HPMC-coated liposomes showed the greater bioadhesion and higher entrapment efficiency than chitosan-coated formulae. The *in vitro* release studies showed prolonged release of sildenafil from coated liposomes as compared to uncoated liposomes and sildenafil solution. *Ex vivo* permeation study revealed the enhanced permeation of coated relative to uncoated liposomes. Chitosan-coated formula demonstrated highest drug permeation and was thus selected for further investigations. Transmission electron microscopy (TEM) and Fourier transform infrared spectroscopy (FTIR) confirmed the successful coating of the liposomes by chitosan. Histopathological *in vivo* testing proved the efficacy of chitosan-coated liposomes to improve blood flow to the vaginal endometrium and to increase endometrial thickness. Chitosan-coated liposomes can be considered as potential novel drug delivery system intended for the vaginal administration of sildenafil, which would prolong system's retention at the vaginal site and enhance the permeation of sildenafil to uterine blood circulation.

## Introduction

Uterine endometrial thickness is a critical factor influencing the successfulness of embryo implantation following *in vitro* fertilization. An endometrial thickness of greater than 8–9 mm at the time of the HCG (human chorionic gonadotropin) trigger, correlates well with an optimal chance of a successful pregnancy, while a lining of less than 8 mm is associated with a poor prognosis (Gonen & Casper, 1990; Sher et al., 1991). Efforts to improve the development of the endometrial lining through administration of estrogen supplementation, low-dose aspirin or administration of high dosage gonadotropin fertility drugs yielded disappointing results (Sher et al., 1993). A marked improvement in estrogen-induced endometrial growth was achieved by nitroglycerin skin patches; however, many women experienced unpleasant side effects during the first few days of treatment (Smith & Brien, 1998). Sher and Fisch (2000) reported that sildenafil, which was proven to increase penile blood flow, showed a great development in uterine lining with subsequent healthy, normal pregnancies without the bothersome side effects of nitroglycerin.

Sher and Fisch (2002) recommended vaginal administration of sildenafil because when absorbed vaginally, it immediately enters the uterine blood circulation in a high concentration and is thus able to improve blood flow and estrogen delivery to the inner endometrial lining. From the uterine lining, sildenafil then immediately passes into the systemic blood circulation in a very low concentration and thus rarely causes any side effects or complications. Conversely, when administered orally, sildenafil is absorbed from the upper gastrointestinal tract, reaches the systemic circulation in a relatively high concentration and can thus cause side effects. A further advantage for the vaginal administration of sildenafil is its moderate oral bioavailability. Despite the high oral absorption of the drug, which reaches up to 92%, the oral bioavailability was found to be only about 40% due to first pass metabolism (Nichols et al., 2002).

However, the market lacks a vaginal dosage form of sildenafil and all researches were carried out on the commercial oral formulation of the drug (Viagra®), which was administered vaginally four times daily to reveal an effective therapeutic effect (Sher & Fisch, 2002). The frequent administration is probably because sildenafil possesses the least lipophilicity and permeability at vaginal pH (Wang et al., 2008) in addition to the rapid clearance of the tablet by the vaginal fluids. The development of a vaginal dosage form that improves the bioavailability of sildenafil is thus of great importance.

Conventional vaginal dosage forms (solutions, suspensions, gels, ovules, creams) are of low cost and easy to formulate but suffer from poor bioavailability. Recent advances in vaginal drug delivery systems are based on encapsulating the drug in micro- or nanocarriers. Among drug carriers used for vaginal delivery are liposomes. Liposomes have the distinct advantages of being both nontoxic and biodegradable because they are composed of phospholipids (e.g. phosphatidylcholine and egg phosphatidylethanolamine) and cholesterol, which are naturally occurring substances. The unique ability of liposomes to entrap drugs in both an aqueous and a lipid phase make such delivery systems attractive for hydrophilic and hydrophobic drugs. Liposomal drug delivery systems also have other favorable properties, including their similarity to the biological membrane and the ability to improve undesirable drug properties such as low solubility and poor membrane permeability (Sharma & Sharma, 1997; Lian & Ho, 2001). Therefore, liposomes have gained a great deal of attention as potential drug delivery systems to maximize drug efficacy (Law et al., 2000). In a study done by Berginc et al. (2012), curcumin was applied in liposomes to the vaginal mucosa. The concentration of curcumin in different layers of vaginal tissue was found to be significantly higher compared to curcumin applied in solution.

However, the effect of gravity and the clearance of the vaginal fluids may result in loss of the liposomal formulation and hence reduced drug efficacy (Woolfson et al., 2000). For this reason, researchers have focused their attention on the development of new systems that can increase the time of permanence of formulations in the vaginal area, basically by using hydrophilic bioadhesive polymers (Valenta 2005), such as polyacrylic acids, cellulose or polysaccharides derivatives that are able to increase, significantly, the retention time on the vaginal mucosa. In addition, coating the liposomes with polymeric coats improves the physical and chemical stability of liposomes presumably by the mechanism of steric stabilization and reduces the leakage of encapsulated material (Sunamoto et al., 1983).

Therefore, the aim of the present study was to incorporate sildenafil into liposomal carrier in order to improve its vaginal bioavailability. Furthermore, liposomes were coated with bioadhesive polymers with the purpose of enhancing bioadhesion, thus increasing the permanence of the drug at site of application.

## Materials and methods

### Materials

Sildenafil citrate was provided by Unipharm (Cairo, Egypt). Lipoid S 100 (PC, soybean lecithin,  > 94% phosphatidylcholine) and cholesterol were obtained from Medical Union Pharmaceuticals (Cairo, Egypt). Hydroxypropyl methylcellulose (Methocel K15M) was purchased from Colorcon (Dartford, United Kingdom). Chitosan (above 85% deacetylation) was obtained from Winlab (Market Harborough, United Kingdom). All other reagents were of analytical grade.

### Methods

#### Preparation of liposomes

Three liposomal formulae (Table 1) were prepared by thin film method (Maestrelli et al., 2006). In brief, 200 mg of phospholipid and cholesterol were dissolved in 10 ml 1:1 (v/v) mixture of chloroform and methanol in a round bottom flask. The organic solvent system was slowly removed under reduced pressure, using a rotary evaporator (RE200, BiBy Sterlin, U.K.) at 40 °C, such that a very thin film of dry lipid was formed on the inner surface of the flask. The remaining film was then re-dispersed in 10 ml of 0.1% w/v sildenafil solution in simulated vaginal fluid (SVF) pH 4.2 (Marques et al., 2011). For size reduction, the liposomal dispersions were placed in ultrasonic bath (LC 60/H, Elma, Germany) for 1 h and then stored in the refrigerator overnight. After 24 h, the liposomal suspensions were centrifuged at 13 500 rpm and 2 °C in a cooling ultracentrifuge (3 K 30 Sigma, Germany) for 1 h. The supernatant was then removed and the cake was redispersed in 10 ml SVF.

**Table 1.** Composition and characterization of different liposomal formulations (mean ± SD, *n* = 3).

	Composition (molar ratio)								
Formula	Lipoid S 100	Cholesterol	Polymer coat	PS (nm)	PDI	Zeta potential (mV)	EE (%)	Bioadhesion (dyne/cm^2^)	*k*
F1	1	1	–	2632 ± 44	0.26 ± 0.13	−7.75 ± 1.2	66.36 ± 3.4	ND^a^	ND
F2	7	4	–	1118 ± 53	0.37 ± 0.05	−0.45 ± 0.7	78.11 ± 5.1	ND	ND
F3	9	1	–	940 ± 25	0.21 ± 0.02	4.14 ± 1.5	89.68 ± 2.6	1705.01 ± 24.5	19.49 ± 0.7
F3C	9	1	Chitosan	1676 ± 75	0.40 ± 0.04	25.11 ± 2.3	72.24 ± 1.2	2697.75 ± 68.7	30.78 ± 3.4^b^5.81 ± 1.4^c^
F3H	9	1	HPMC	2648 ± 32	0.62 ± 0.11	19.52 ± 1.1	78.39 ± 4.5	4905.23 ± 55.3	5.33 ± 0.5

^a^Not determined.

^b^ Initial phase of release profile.

^c^ Second phase of release profile.

#### Coating of liposomes

The sildenafil containing liposomal formulation (F3) was coated with 0.6% (w/v) chitosan solution in 1% glacial acetic acid (F3C) and with 0.6% (w/v) HPMC aqueous solution (F3H). An equal volume of polymer solution was added dropwise to liposomal dispersion under magnetic stirring at room temperature for 1 h. The coated liposomes were then placed in refrigerator overnight for stabilization.

#### Characterization of liposomes

##### Particle size and polydispersity index

The mean particle size and size distribution of freshly prepared liposomal dispersions were determined using a Zetasizer (2000) (Malvern Instruments Ltd., UK). Light scattering was monitored at a 90° angle and a temperature of 25 °C. The measurements were performed after diluting samples by 100-fold with SVF at ambient temperature. The predetermined refractive index of the different formulae was incorporated into the computer software of the Zetasizer, which calculated the mean size and polydispersity from intensity.

##### Zeta potential

The zeta potential was determined using a Zetasizer (2000, Malvern Instruments Ltd., UK). The liposomal dispersion (0.25 ml) was diluted with 0.001 M KCl and placed in the electrophoretic cell. The zeta potential values were calculated from the mean electrophoretic mobility values.

##### Entrapment efficiency (EE %)

The amount of drug entrapped in the liposomes was determined by diluting freshly prepared sildenafil-loaded liposomal suspension 1:100 with methanol. The clear solution was analyzed for the free drug spectrophotometerically at 292 nm (UV-1650 P.C; Shimadzu, Japan) against a blank of plain liposomes treated by the same method. The experiment was performed in triplicate. The percent entrapment efficiency of drug was assessed by comparing the amount of entrapped drug to total amount of drug initially present in the liposomal dispersion.

##### In-vitro drug release study

The *in vitro* release test was performed using an oscillating water bath (LSB-015S, Daihan Labtech, Korea). Samples of 3 ml of sildenafil-loaded liposomal suspensions were placed in glass cylindrical tubes (2.5 cm in diameter and 10 cm in length) with one closed end and the other end tightly covered with a cellulose membrane soaked overnight in SVF pH 4.2. The cylindrical tubes were held by a holder so that the membrane-covered ends were immersed in 75 ml SVF (pH 4.2). The release study was carried out at 37 ± 0.5 °C at a shaking speed of 50 rpm. Aliquots of 3 ml were withdrawn at different time intervals and replaced each time with fresh SVF. The withdrawn samples were analyzed for sildenafil by measuring the absorbance at 292 nm (UV spectrophotometer; UV-1650 P.C Shimadzu, Japan) against the samples withdrawn at respective time interval from plain liposomal dispersions treated by the same manner. The experiment was performed in triplicate and the percentage released of sildenafil was calculated.

##### Drug release kinetics

Data obtained from the drug release study were fitted into zero order, first order, Higuchi and Korsmeyer–Peppas models to predict the drug release mechanism from the studied formulae.

##### Ex-vivo transvaginal permeation

Transvaginal permeation study of the liposomal formulations was carried out as previously described in the release study using fresh bovine vaginal mucosa as membrane instead of cellulose acetate membrane. The tubes used in the study were smaller (d = 0.5 cm) than those used for *in vitro* release experiment so that the vaginal mucosa available for drug permeation was 0.78 cm^2^. The receptor compartment was filled with 5 ml PBS (pH 7.4) and was kept at 37 ± 0.5 °C with constant shaking at 50 rpm. Permeation study continued for 24 h and samples were withdrawn at predetermined time intervals and were analyzed for sildenafil by HPLC. An HPLC assay reported by Tripathi et al. (2013) for the determination of sildenafil was adopted. The mobile phase was composed of methanol and deionized water in a ratio of 85:15 v/v. Separation was performed on a 4.6 mm × 150 mm, 5 μm, Zorbax SB-C18 column (Agilent Technologies, Santa Clara, CA). The mobile-phase flow rate was 1 ml/min. The system was operated at ambient temperature and the detection wavelength was 230 nm (Isocratic pump, IC-10AS, UV/VIS detector; SPD 10A, Shimadzu, Japan). The sample volume was 10 μl.

##### Bioadhesion study

A modified balance method was used to determine the bioadhesive performance of the liposomal formulations (Choi et al., 2003). The instrument is broadly composed of a modified two arm physical balance in which the left pan had been replaced by a 100 g weight, which was suspended by means of a thin steel wire. A section of fresh bovine vaginal tissue obtained from a local slaughterhouse, was washed with normal saline and was attached to the lower flat surface of the 100 g weight by cyanoacrylate adhesive. On the right pan, an empty beaker was placed and then the balance was tarred. Beneath the 100 g weight, an inverted beaker was placed onto which another piece of vaginal tissue was glued. One milliliter of liposomal formulation was added onto the bovine vaginal tissue of the inverted beaker at room temperature. The 100 g weight was removed from the steel wire of the left side of the balance and was left in contact with the formulation on the inverted beaker for 4 min. Then, the 100 g weight was reattached and water was dropped in a constant flow rate of 13–15 drops per minute by means of an infusion apparatus. The weight of the water in the beaker was kept increasing until the vaginal tissues just detached.

The minimal weight of water required to detach the sample from the vaginal mucosa was recorded. The experiment was repeated three times in an identical manner (*n* = 3). The bioadhesive force, that is, the detachment stress (dyne/cm^2^) was determined using the following equation (Ch'ng et al., 1985):

$$\mathrm{Detachment}\;\mathrm{stress}\;(\mathrm{dyne}/{\mathrm{cm}}^{2})\;=\;\frac{m\times g}{A}$$

where *m* is the weight of water, *g* is the acceleration due to gravity taken as 981 cm/sec^2^ and A is the area of contact of cow vaginal tissue.

##### Fourier transform infrared (FTIR) spectroscopy

FTIR spectra of the drug, pure chitosan and lyophilized samples (Freezone lyophilizer; Labconco Corporation) of uncoated liposomes and chitosan-coated liposomes deposited in KBr disks were recorded on a Jasco FT/IR 460 plus (Japan) spectrometer. The scanning was done in the range of 400–4000 cm^−1^ with a speed of 2 mm/s at a resolution of 4 cm^−1^ at room temperature. The band width was measured at 50% of height of the peaks.

##### Transmission electron microscopy (TEM)

Morphology of liposomes was characterized using TEM. (JOEL JEM-1230, Japan) operating at 200 kV capable of point-to-point resolution. Combination of bright field imaging at increasing magnification and of diffraction modes was used to reveal the form and size of the liposomes. In order to perform the TEM observations, the liposomal dispersion was diluted with SVF (1:10). A drop of the diluted liposomal dispersion was applied to a copper grid coated with carbon, and the excess was drawn off with filter paper. The sample was stained by 1% aqueous solution of phosphotungestic acid for 2 min, left to dry and then examined under transmission electron microscope.

##### Histopathological study

(1) Animal housing and handling: All experiments were approved by the Institutional Animal Ethics Committee, Cairo University, Egypt and they comply with the ARRIVE guidelines. Thirty female mature Albino rats with an average weight of 180–200 g were obtained from the experimental animal care center, Faculty of Veterinary Medicine, Benha University. They were kept in rodents' cages at a temperature of 22 ± 1 °C and relative humidity of 55 ± 5%. Water and rodents' chow were provided *ad lib*. The animals were kept in a dark:light cycle of 12 h each. Rats were left for 7 days for adaptation before the beginning of experiment. The female rats were divided into three groups of 10 rats each. The first group was used as control and the second group was given 0.28 mg/kg body weight/day sildenafil citrate solution by intravaginal administration using a metallic tube twice daily for 16 days and the third group received 0.28 mg/kg body weight chitosan-coated liposomal dispersion intravaginally by the same manner and duration like group 2.

(2) Tissue collection and fixation: After 16 days, all rats were sacrificed and dissected carefully to take the female genital tract. The vaginal tissues were fixed in 10% formalin solution for 1 week. Specimens from different parts of the female genital tract were dehydrated in ethyl alcohol, cleared in xylol and embedded in paraffin. Sections of 5 μm were cut and stained with Periodic acid Schiff (PAS) for histopathological examination.

##### Statistical evaluation

The Student's *t*-test was used for comparison of two means. A significance level of *p* < 0.05 was considered to be appropriate.

## Results and discussion

### Characterization of uncoated liposomes

In the present study, three phosphatidyl choline/cholesterol ratios were studied with respect to zeta potential, particle size, entrapment efficiency and polydispersity index. The change in cholesterol content was found to alter the characteristics of liposomal vesicles significantly.

#### Zeta potential

Liposomes may acquire a negative, positive or neutral charge according to the type of lipid used in the preparation (Brgles et al*.,* 2008). Although, in the present study, liposomes were prepared with phosphatidylcholine, which is a neutral lipid, they possessed a slight negative charge at higher cholesterol ratios and a slight positive charge at low cholesterol content (Table 1). This finding is consistent with the results obtained by Garcia-Manyes & Sanz (2005), who reported that phosphatidylcholine bilayer acquired negative zeta potential values in ultrapure water probably due to hydration layers formed around the surface (Egawa & Furusawa, 1999) and to the orientation of lipid headgroups (Makino et al., 1991). However, they observed a shift of zeta potential to positive values upon increasing the ionic strength of the medium, which was attributed to membrane interaction with mono- and divalent cations present in the medium. Magarkar et al. (2014) studied the effect of cholesterol on the interaction between the lipid bilayer and Na^+ ^cations present in the surrounding physiological medium. They found that Na^+ ^ cations bind dominantly to phosphate oxygen of phospholipids, in contrast to a very small level of Na^+  ^binding to the cholesterol oxygens. Therefore, increasing the level of cholesterol in the lipid membrane reduces Na^+ ^ binding to lipid head groups, which reduces the surface charge of the membrane. On the other hand, the increase in the level of cholesterol increases the hydrophobicity of the membrane, which further decreases the membrane affinity for cations.

#### Particle size and polydispersity index

Liu et al. (2000) postulated that incorporating cholesterol into the bilayer causes the lipid vesicles to change their packaging geometrical structures, which include lipid vesicle size, the curvatures of surface bilayer and surface bilayer rigidity. From Table 1, it is obvious that, the increase in cholesterol content resulted in an increase in liposomal size (*p* < 0.01). This is in agreement with the finding of Sułkowski et al. (2005) who found that the presence of cholesterol in liposomal membrane causes an increase in the distance between the phospholipid chains and limits the possibility of interaction between electronic shells of polar head groups of phospholipids in the bilayer. Moreover, it is reported that the effect of the amount of cholesterol on the size of the liposomes is greater at pH 4, which is closer to the isoelectric point of phosphatidylcholine containing liposomes (Petelska & Figaszewski, 1973).

The polydispersity index (PDI) is a measure of the width of unimodal size distributions (Verma et al., 2003). An acceptable polydispersity index should have the value below 0.7. The polydispersity indices of the prepared liposomes were in general small, which indicates a good dispersion homogeneity.

##### Entrapment efficiency

**Figure 1.**
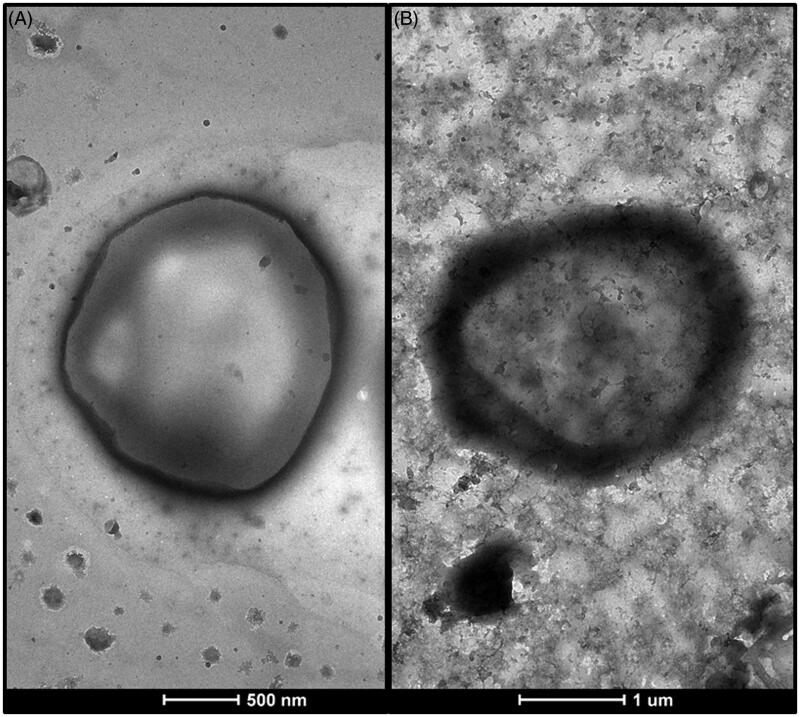
Transmission electron micrographs of A: uncoated liposomes (F3), B: chitosan-coated liposomes (F3C).

Many factors may influence the entrapment efficiency of drugs in liposomes such as liposome size and type, charge on the liposome surface, bilayer rigidity, method of preparation and characteristics of the drug to be encapsulated (Kulkarni et al*.,* 1995). Hydrophobic drugs are solubilized in the phospholipid bilayer of the liposomes and once entrapped they remain in the liposome bilayer as they have very low affinity toward the inner or outer aqueous regions of the liposomes that generally results in high entrapment efficiencies. A hydrophilic drug may not be encapsulated with high efficiency because the drug molecules can diffuse in and out of the lipid membrane. Thus, the drug would be difficult to retain inside the liposomes (Çağdaş et al., 2014). In some cases, the hydrophobicity or hydrophilicity of the drug depends on the pH of the aqueous medium as in the case of sildenafil citrate. This is because sildenafil is an amphoteric compound and can adopt positive or negative charge, depending on the pH of the medium (Gobry et al., 2000). Thus, any change in pH causes a change in the aqueous solubility of the drug and thus affects its partitioning. Wang et al. 2008 showed that, the cationic species of sildenafil exist at acidic pH, anionic species at alkaline pH, whereas unionized molecules at neutral pH. The pH solubility profile of sildenafil showed highest water solubility at pH 3–4, which reduces rapidly upon increasing the pH to neutral value. Ahmed (2014) investigated the entrapment efficiency of sildenafil in transfersomes. Results showed that, as the pH of the hydration medium increased (5.5–7.5), the solubility of the drug in the hydration medium decreased, which led to migration of the drug into lamellar layers revealing high entrapment efficiency. This result disagrees with the results obtained in the present study. The entrapment of sildenafil was relatively high despite the higher solubility of the drug at pH 4.2 (Table 1). This finding may be attributed to the type and size of formed vesicles. Kulkarni et al. (1995) reported that the encapsulation of hydrophilic molecules depends mainly on the volume of the aqueous phase encapsulated during liposome formation. Thus, the greater the entrapped aqueous volume, the larger would be the encapsulation efficiency of the hydrophilic drug. The TEM micrograph of prepared liposomes (F3) shows clearly a large central liposome volume (Figure 1), which agrees with the high entrapment efficiency of the drug. Similar results were obtained by Naeem et al. (2016), who found that the encapsulation of hydrophilic crystal violet was much greater than that of the hydrophobic Nile red due to large central aqueous compartment of the liposomes. Furthermore, the positive charge carried by the drug at the acidic pH of SVF (pH 4.2) may be another reason for the high encapsulation of sildenafil. It is reported by Çağdaş et al. (2014) that, ionized drug molecules, once entrapped inside the liposome, lose their ability to diffuse out of the lipid membrane, which results in high concentration of ionized drug inside the liposome.

Sildenafil loading to liposomes decreased with an increase in cholesterol concentrations (Table 1). The inclusion of cholesterol in the liposome may affect the membrane organization, dynamics and vesicle diameter, which consequently affect the encapsulation efficiency of the drug (Taylor et al., 1990). Glavas-Dodov et al. (2005) reported that, when cholesterol was added into the liposomes, the encapsulation efficiency of 5-fluorouracil and ampicillin was decreased due to a reduction in the internal liposome volume. Deniz et al. (2010) also postulated that, cholesterol might locate itself close to the glycerol backbone of the membrane resulting in a denser and more rigid bilayer, which lowers the chance of the drug molecule to be encapsulated into the liposome.

### Characterization of coated liposomes

Based on the characteristics of the liposomal formulations with different phosphatidylcholine/cholesterol ratios, F3 was selected for its high entrapment efficiency, high dispersion homogeneity and small particle size to be coated with chitosan and HPMC to study the effect of coating on the physicochemical properties of liposomal dispersions. The selection of the polymers was based on their biodegradability, biocompatibility and reported mucoadhesive properties (Lehr et al., 1992; Akbari et al., 2010). The mechanism of coating of the polymers to the nearly neutral liposomes probably involved mainly hydrogen bonding between the polysaccharide and the phospholipid head groups (Guo et al., 2003) as well as hydrophobic attraction of the hydrophobic segments of the polymer chain with the lipidic bilayer (Lockhead, 1992). It was also reported that, when cholesterol was included as a component of the bilayer it would allow a hydrophobic interaction at the surface level of the vesicle (Bernsdorff et al., 1997). Furthermore, Jøraholmen et al. (2014) postulated that although liposomes are nearly neutral, the electrostatically driven binding of chitosan to the lipid membrane is energetically favored.

#### Particle size, zeta potential, PDI and entrapment efficiency

Coating with different polymers increased the original size of the vesicles significantly (*p* < 0.001) (Table 1), which confirms the successful coating of liposomes. HPMC-coated liposomes (F3H) possessed larger particle size than chitosan-coated liposomes (F3C). Based on the belief that, the molecular weight and viscosity of the polymeric solution may affect the thickness of the polymer coat, the viscosity of the polymeric solutions of HPMC and chitosan was measured) Cone and plate viscometer; model III, Brookfield, WI) at minimum shear (50 rpm) and was found to be 89.9 ± 5.5 and 13.6 ± 2.5 dyne/cm^2^, respectively. A higher molecular weight is accompanied by an increased number of binding sites between polymer chain and liposomal bilayer, which results in a thicker polymeric coat.

Coated liposomes showed a reduced entrapment efficiency in comparison to uncoated liposomes, which indicates that some drug have escaped the liposomes during the coating process. Higher entrapment efficiency was observed for HPMC-coated liposomes probably due to greater thickness of the polymeric coat, as it is noticeable from Table 1 that the increase in entrapment efficiency was consistent with particle size enlargement.

Furthermore, the electrostatic repulsion between the positively charged drug and the cationic chitosan could explain the lower entrapment efficiency of the chitosan-coated liposomes.

The formation of a thick polymer layer on the bilayer’s surface also influenced greatly the zeta potential of coated liposomes. From Table 1, it is to be noticed that there was a remarkable increase in zeta potential upon coating for all formulae. Since chitosan carries a positive charge, the highest zeta potential was observed for chitosan-coated liposomes. The positive charge carried by the drug at vaginal pH may explain the increase in zeta potential of HPMC-coated liposomes despite being a neutral polymer. This increase in zeta potential would lead to a more stable colloidal dispersion due to greater repulsion between particles and reduced tendency for the particles to come together (Paolino et al., 2006).

#### Bioadhesion study

The prerequisite for successful topical vaginal therapy is the prolonged contact of drug-containing formulation with the vaginal surface. In order to evaluate the residence time of liposomes on the vaginal tissue, the bioadhesive performance of uncoated and polymer-coated liposomes on excised bovine vaginal mucosa was investigated. As expected, coating the liposomes with chitosan or HPMC improved remarkably the bioadhesion of the liposomal formulations (*p* < 0.001) (Table 1).

It is previously reported that, the degree of bioadhesion depends on type and amount of polymer, degree of hydration, polymer chain length, molecular weight of polymer, degree of interpenetration of polymer chains and type of bonding between polymer chains and mucin (Andrews et al., 2009). The bonding of bioadhesive polymers to biological membrane occurs mainly through hydrophobic interactions, hydrogen bonding, van der Waals bonds and ionic interactions (Woodley 2001).

The most important mucoadhesive mechanism of chitosan is the ionic interaction between the positively charged amino groups of the polymer and the negatively charged sialic acid residues of mucus gel layer (Lehr et al., 1992). Chitosan possesses also OH and NH_2_ groups that can give rise to hydrogen bonding (Valenta, 2005). At low pH, the molecules become more ionized, are uncoiled, possess high charge density and assume a more elongated shape. Hence, at low pH values, for example, vaginal pH, chitosan has better chances for an intimate contact with the epithelial membrane and for the electrostatic interaction with the anionic component (sialic acid) of the glycoproteins of the epithelial cells. However, results show significant superior bioadhesive strength for HPMC-coated liposomes compared to chitosan-coated liposomes (Table 1). HPMC is a nonionic hydrophilic polymer, the mucoadhesive performance of which relies mainly on the interpenetration of polymer chains and mucin molecules and on the formation of strong hydrogen bonding (Jaipal et al*.,* 2013). The greater mucoadhesive performance of HPMC is probably attributed to its high molecular weight, which was indicated by the high viscosity of the polymer solution. It is reported that the mucoadhesive strength of a polymer increases with increasing molecular weight due to deeper interpenetration of polymer and mucus chains in addition to the increase in number of polar functional groups on the molecular chain capable of forming hydrogen bonding with mucin (Jiménez-Castellanos & Zia, 1993). Unlike chitosan, HPMC being nonionic, the bioadhesive properties of which, is not affected by the pH of the medium.

#### *In vitro* drug release study

**Figure 2.**
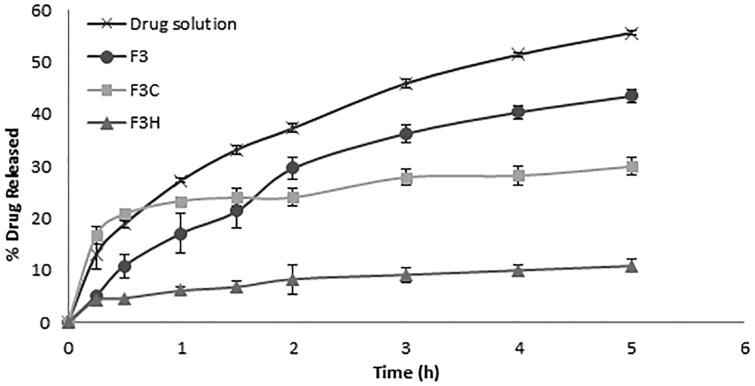
Percent of drug released from drug solution, uncoated liposomes (F3), chitosan-coated liposomes (F3C) and HPMC-coated liposomes (F3H) (*n* = 3).

Figure 2 shows the release profiles of sildenafil from uncoated and coated liposomes in comparison to drug solution. It is obvious that the encapsulation of the drug into liposomal formulation significantly slowed its release indicating the depot effect of liposomes (*p* < 0.01). Kinetic analysis of *in vitro* release data revealed that the release profiles of all liposomal formulae followed square root time-dependent Higuchi model, which is described as a diffusion process based on Fick's law. In order to interpret the difference of the release behavior of various formulations, the release rate constant (*k*) was calculated (Table 1). The release constant (*k*) of uncoated liposomes (F3) was significantly higher than that of the coated liposomes (*p* < 0.05), indicating that coating provided a retention effect on sildenafil release. This is probably because the coating layer constructs an intense shell on the liposome surface and restricts the fluidity of lipid bilayer, which leads to a decrease in the membrane permeability (Li et al., 2009). The slowest release rate was shown by HPMC-coated liposomes (F3H). This might be due to the relatively low rate of corrosion of high MW HPMC coating layer. Moreover, the greater particle size of F3H, which indicates a thicker coat, and consequently, a longer diffusion path may have contributed to the slower drug release from this formula compared to F3C. It is worthy to note that chitosan-coated liposomes showed a biphasic release profile with a remarkable boost release at the beginning of the study. The rate constant was thus calculated for each phase separately. The initial fast release, characterized by a relatively high *k* value, may be attributed to fast detachment of drug molecules that were only loosely bound to liposomal surface. Since the drug carries a positive charge at acidic pH, the repulsive forces between the cationic polymer and the cationic drug were probably strong enough to overcome the attractive forces resulting in a facilitated separation of the drug from liposomal surface. The second slow release phase was probably governed by the slow corrosion of the coating layer.

#### *Ex vivo* transvaginal permeation

**Figure 3.**
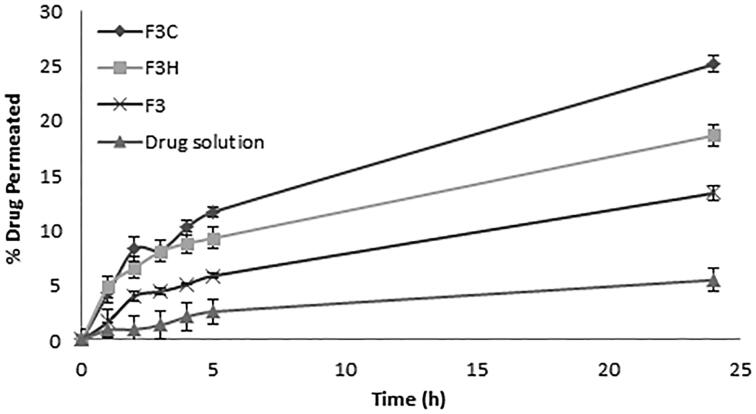
Percent of drug permeated through excised bovine vaginal mucosa from drug solution, uncoated liposomes (F3), chitosan-coated liposomes (F3C) and HPMC-coated liposomes (F3H) (*n* = 3).

A permeation study from drug solution and liposomal formulations through bovine vaginal mucosa was performed. In contrast to *in vitro* release study, the permeation of sildenafil through vaginal mucosa has shown to be least for drug solution and highest for polymer-coated liposomes (*p* < 0.001) (Figure 3). Wang et al. (2008) investigated the pH-dependent solubility and *in vitro* transmucosal permeability of amphoteric sildenafil. The researchers found that among the other species of the drug (anionic and neutral) the cationic species, which exist at acidic pH, possess highest solubility and least membrane permeability. Gobry et al. (2000) reported that the high lipophilicity of the cationic form of the drug is due to its capacity to form intramolecular H-bonds between one O atom of the sulfonamide function and the proton of the basic N atom. This could explain the low permeation of the drug solution and shows the importance of encapsulating the drug in liposomal system.

Polymer coating modifies the surface characteristics of liposomes regarding the mucoadhesive properties and the surface charge. The profound greater mucoadhesive characteristics of polymer-coated liposomes improves the retention of the liposomes at the membrane surface giving higher chance for the drug to pass through the membrane. Furthermore, the negatively charged lipid present in the lipid layer of the vaginal mucosa could interact with the positively charged polymer-coated liposomes, resulting in an increased permeation of the drug through the membrane. Despite of the greater mucoadhesive property of HPMC-coated liposomes, a significantly higher permeation was observed for chitosan-coated liposomes (*p* < 0.01) at 24 h (Figure 3). This result indicates that the chitosan-coated liposomal system was superior for the transvaginal delivery of sildenafil. In addition to the higher zeta potential of chitosan-coated liposomes and the smaller thickness of the polymer coat, chitosan is known to possess permeation enhancing properties for hydrophilic drug molecules due to its ability to open epithelial tight junctions by structural reorganization of the proteins. This allows a greater paracellular transport of drug molecules (Borchard & Junginger, 2001; Bravo-Osuna et al*.,* 2007). Based on the results obtained from the *ex vivo* transvaginal permeation study, chitosan-coated liposomal formulation was selected for further studies.

#### Transmission electron microscopy (TEM)

The morphology of uncoated liposome and chitosan-coated liposome (F3C) was examined by transmission electron microscopy (Figure 1). The TEM image reveals that the vesicles were almost spherical. In addition, the existence of chitosan surrounding the liposomes was well visualized on the surface of chitosan-coated liposomes and resulted in an increase in liposomal size.

#### Fourier transform infrared spectroscopy (FTIR)

**Figure 4.**
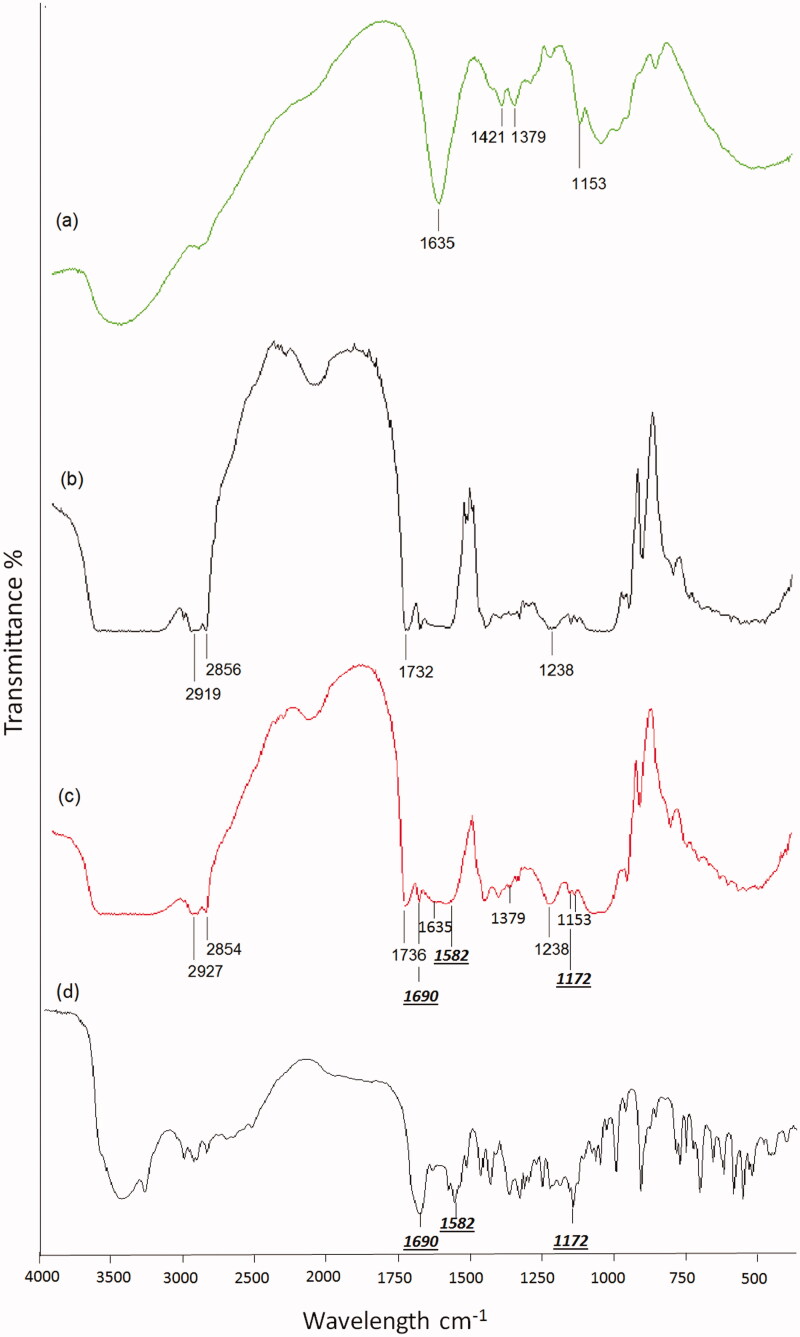
FTIR spectra of (a) chitosan, (b) uncoated liposomes (F3), (c) chitosan-coated liposomes (F3C) and (d) sildenafil citrate.

Figure 4(a) shows the FTIR spectrum of pure chitosan. The main bands appearing in that spectrum are due to bending vibrations of methylene and methyl groups at 1379 cm^−1^ and 1421 cm^−1^, respectively. Absorption at 1635 cm^−1^ is related to the vibrations of carbonyl bonds (C = O) of the amide group CONHR (secondary amide). The band located at ν = 1153 cm^−1^ is related to asymmetric vibrations of CO in the oxygen bridge resulting from deacetylation of chitosan (Paluszkiewicz et al., 2011). Most of the characteristic bands of chitosan could be detected in the FTIR of chitosan-coated liposomes (Figure 4c). FTIR spectroscopy was also used to monitor subtle changes in the structure of the lipid assemblies by analyzing the frequency and the bandwidth of different functional groups and by investigating the acyl chains and head-group region of the lipid molecule in the presence (Figure 4c) or absence of chitosan (Figure 4b). In the analysis of the FTIR spectra, the symmetric and antisymmetric CH_2_ (at 2800–3000 cm^−1^) bands, the C = O (at 1735 cm^−1^) stretching bands and the PO_2_^− ^ (at 1220–1240 cm^−1^) antisymmetric double stretching bands of uncoated and chitosan-coated phosphatidylcholine liposomes were investigated.

From Figure 4(c), it is to be noticed that the band at 2856 cm^−1^, due to symmetric CH_2_ stretching modes of the acyl chains, was slightly shifted to lower frequency. However, the broad band at ∼2920, due to antisymmetric CH_2_ stretching, was slightly increased to higher frequencies. This is generally attributed to an increase of the acyl chains disorder, and thus to an increase in the average number of gauche bonds (Dicko et al., 1999). Moreover, the bandwidths of the CH_2_ stretching bands give dynamic information about the system (Severcan et al*.,* 2005). The band width of antisymmetric CH_2_ stretching band, for example, at 2919.5 cm^−1^, decreased from 70 to 33 cm^−1^ for chitosan-coated liposomes, which implies a decrease in the membrane fluidity and thereby stabilization of the system in the gel phase (Biruss et al., 2007).

In order to analyze the interaction of chitosan with the glycerol backbone near the head group of the phospholipids, the C = O stretching band was analyzed. In contrast to a study done by Liu et al., (2013) the C = O band at 1732 cm^−1^ was shifted by the presence of chitosan to higher frequency (1736 cm^−1^), indicating the absence of hydrogen bonding and the existence of free carbonyl groups in the system. This finding may indicate that chitosan decreases the strength of hydrogen bonding in the interfacial region of the bilayer. It is possible that chitosan displaces some H_2_O molecules from the interfacial region resulting in an increase in the number of free carbonyl groups (Toyran & Severcan, 2003). The interaction between chitosan and the head group of the lipids was investigated by the observation of the PO_2_^− ^ antisymmetric double stretching band at 1238 cm^−1^. No change in the frequency of the band could be detected; however, the bandwidth decreased from 60 to 40 cm^−1^. The narrowing in the bandwidth indicates that mobility of the head group decreases (Choi et al., 1991). FTIR results demonstrate that chitosan has been successfully coated on the surface of liposome.

Furthermore, observing the FTIR spectra of uncoated (Figure 4b) and coated liposomes (Figure 4c), characteristic peaks of the drug (Figure 4d) could be detected including a peak at 1172 cm^−1^ corresponding to the SO_2_ group in addition to two bands at 1580 and 1690 cm^−1^ analogous to the aromatic C = C bond and the carbonyl group of the pyrazole ring, respectively.

#### Histopathological study

**Figure 5.**
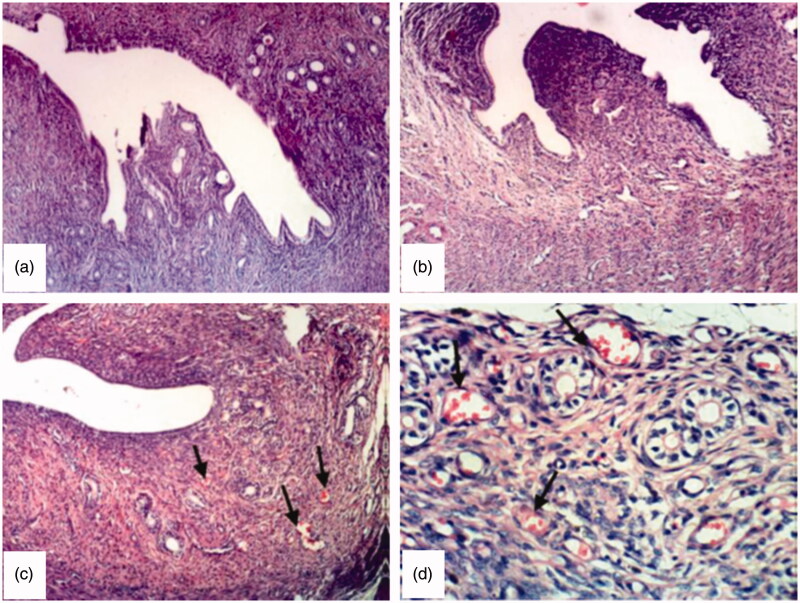
Photomicrograph of uterus from (a) group 1, control, untreated rat (×100), (b) group 2, rat treated with sildenafil solution (×100), (c) group 3, rat treated with chitosan coated liposomal dispersion, arrows show dilated and congested endometrial blood vessels, which appear as red spots (×100) (d) same as (c) at higher magnification (×400).

Figure 5(a) shows the normal histological structure of uterine layers (endometrium, myometrium and perimetrium) of untreated rat (group 1, control). Examined sections from group 2, which received drug solution, revealed no significant histopathological changes (Figure 5b). However, sections from group 3, which were treated with chitosan-coated liposomal dispersion, revealed a clear dilatation and congestion of endometrial blood vessels (Figures 5c and d) in addition to an increase in the thickness of endometrial layer from 154.7 ± 3.7 μm (control rats) to 207.6 ± 0.6 μm. This finding confirms the poor availability of the drug solution to endometrial tissue, which may be on one hand due to the poor retention of the drug solution and its rapid clearance from vaginal surface, and on the other hand due to the low permeability of the drug in its cationic form. Normal microflora predominantly lactobacilli produce sufficient lactic acid to acidify vaginal secretions to pH 3.5–4.5 (Stewart-Tull, 1964; Boskey et al*.,* 2001), which significantly lowers the permeation of the drug (Wang et al., 2008). However, the incorporation of the drug into chitosan-coated liposomes significantly improved its effect. This finding could be explained by the strong bioadhesive properties of chitosan, which increase the residence time of liposomes on endometrial surface and therefore reduces the vaginal leakage in addition to the permeation enhancing effect of chitosan by promoting the paracellular transport mechanism (Borchard & Junginger, 2001). Moreover, liposomes are known to improve the permeation of hydrophilic molecules through biological membranes. Drug delivery from liposomes is not only achieved via passive diffusion of the drug through the liposomal bilayer and the subsequent diffusion through the cell membrane, but through a number of other transfer mechanisms including the fusion of the liposome to the cell membrane, protein-mediated transfer, or phagocytosis and pinocytosis (Wang et al. 2012). These transfer mechanisms could successfully transport hydrophilic molecules, which would not easily pass the membrane by simple diffusion.

## Conclusion

In this study, a mucoadhesive liposomal formulation of sildenafil for vaginal application was developed. The effectiveness of coating of the liposomes by chitosan and HPMC coatings was confirmed by the prolonged release of sildenafil, the increase in both size and zeta potential of liposomes as well as the significant improvement in their mucoadhesiveness. TEM and FTIR studies also confirmed the successful coating of liposomes. Chitosan-coated liposomes revealed the greatest permeation of sildenafil through vaginal mucus *in vitro* compared to HPMC-coated liposomal dispersion, drug solution and noncoated liposomes. Vaginal application of chitosan-coated liposome to female rats showed a clear dilatation and congestion of endometrial blood vessels with increase in the thickness of the endometrial layer compared with the control and drug solution groups. Therefore, the chitosan-coated liposomal formulation appears to have the potential to improve the vaginal bioavailability of sildenafil. In order to facilitate the vaginal application of the developed formula, a further investigation on its incorporation into a gel or cream is recommended.
